# Cognitive reflection is a distinct and measurable trait

**DOI:** 10.1073/pnas.2409191121

**Published:** 2024-11-27

**Authors:** Andrew Meyer, Yigal Attali, Maya Bar-Hillel, Shane Frederick, Daniel Kahneman

**Affiliations:** ^a^Marketing Department, The Chinese University of Hong Kong Business School, Ma Liu Shui, Hong Kong; ^b^Duolingo, Pittsburgh, PA 15206; ^c^Emerita, Center for the Study of Rationality, Department of Psychology, The Hebrew University of Jerusalem, Mount Scopus 9190500, Jerusalem, Israel; ^d^Marketing Department, Yale School of Management, New Haven, CT 06511; ^e^Emerita, School of Public and International Affairs, Princeton University, Princeton, NJ 08544

**Keywords:** cognitive reflection, individual differences, dual-system theory

## Abstract

We report the findings of an adversarial collaboration examining whether the cognitive reflection test (CRT) measures anything beyond mathematical aptitude and, if so, whether its incremental predictive validity can be attributed to reflection, per se. We found that an 8-item CRT has greater predictive validity than an 8-item Mathematical Aptitude Test (MAT) consisting of comparably difficult items which lack dominant intuitive lures. Further, the incremental predictive validity stems from the CRT’s measurement of reflection, which we show using both structural equation models and a dual-response paradigm that helps distinguish susceptibility to intuitions from inadequate mathematical aptitude.

## Significance

We show that the cognitive reflection test predicts a wide array of beliefs, preferences and judgments better than more typical math tests. We demonstrate that its surplus validity can be entirely explained in terms of the construct it purports to assess, and we illustrate how this construct can be assessed with items that do not involve math.

*It doesn’t matter how smart you are unless you stop and think.*

—Thomas Sowell

The “cognitive reflection test” (CRT) was advanced as a behavioral measure of the “need for cognition”—a way to distinguish those who are inclined to stop and think from those who are not (1). Its items were intended to evoke erroneous intuitions that most would succumb to, but which could be corrected by those who bothered to reflect upon or scrutinize them. It is now widely used to illustrate a “dual-system” view of cognition (2–5), which contrasts rapid, casual, or impulsive responses (“System 1”) with more effortful deliberations (“System 2”).

Of its three items, the “bat & ball” problem below is the best known. Most answer 10 cents, both quickly and confidently (5).

A bat and a ball cost $1.10 in total. The bat costs $1.00 more than the ball.

How much does the ball cost? ____ cents

Although typically completed in less than 2 min, CRT scores predict, among other things, greater patience (1), job performance (6), and desire for explanatory depth (7), and less risk aversion (1), religiosity (8–10), and susceptibility to bullshit (11).

Though the CRT, like any test, requires many cognitive abilities (literacy, numeracy, and intelligence), Frederick (1) cautiously conjectured that it also measures the disposition or ability to reflect, because the tempting “lures” provide an attractive off-ramp for those disinclined to stop and think. This special feature is a plausible explanation for its predictive validity—particularly if what is being predicted also requires, or benefits from, reflection or deliberation. Though some support this account (1, 12, 13), others contend that the CRT’s predictive validity stems only from the mathematical content of its items (14–20).

We engaged in an “adversarial collaboration” (21) to test these competing interpretations, comparing the predictive validity of an 8-item CRT with the predictive validity of an 8-item mathematical aptitude test (or MAT). These items were comparably difficult but lacked the distinguishing feature of CRT items—a dominant intuitive lure which tempts respondents into performing some simple, but inappropriate, operation.^*^

The validity of each test was assessed with respect to four dependent variables: 1) numeracy, 2) beliefs (about evolution, religiosity, the paranormal, and the profundity of aphorisms), 3) preferences (regarding delay or risk), and 4) reflection (in contexts not requiring math). Though we all agreed that these four dependent measures spanned item types for which the CRT’s eponymous construct would be more or less relevant, we disagreed about whether or where we would observe discriminant validity. Our results revealed that the CRT was a worse predictor of numeracy, but a better predictor of everything else.

To test whether the CRT’s surplus predictive validity results from the putative construct of “cognitive reflection,” we conducted two analyses. First, we repurposed our nonmathematical reflection scale as another IV (along with MAT and CRT) and used structural equation modeling to identify the components of CRT’s predictive validity for our remaining DVs. Second, we used a dual response procedure—in which respondents were alerted to their errors and received an unanticipated opportunity to correct them—to assess whether the 2nd chance *increases* the test’s predictive validity (which we would expect if mathematical abilities were the central causal factor) or *reduces* it (if respondents’ disposition to scrutinize their intuitive responses drove these relations).

## Procedure and Materials

We recruited participants from four different online platforms, and, following preregistered restriction rules (https://doi.org/10.17605/OSF.IO/NXKE3), attained a final sample of 4,407. (Details of our sampling procedure can be found in our *SI Appendix* along with a demographic breakdown.) The research protocol was approved by Yale’s IRB (ID # 2000031031) and all participants gave informed consent.

Our 8-item CRT used variants of the three original items (1) and five others we thought would function similarly. It appeared first, with the eight items presented in random order on separate screens. The verbatim wording of each item is shown in Table 1, along with the correct answer, the most common error, and respective frequencies of those two responses.

**Table 1.** An 8-item CRT

	Item	Solution _Rate_	MCE _Rate_
**1.**	A drill and a hammer cost $330 in total. The drill costs $300 more than the hammer. How much does the hammer cost? $ ___	15 _26%_	30 _67%_
**2.**	A dog and a cat weigh 100 pounds in total. The dog weighs 86 pounds. What is the difference in weight between the dog and the cat? ___ pounds	72 _40%_	14 _54%_
**3.**	After hatching from its egg, a baby bird doubles in weight every day. On day 12 it weighs a pound. When did the bird weigh half a pound? On day ___	11 _55%_	6 _29%_
**4.**	When it’s on sale for 20% off, a toaster costs $100. When it’s not on sale, how much does it cost? $ ___	125 _37%_	120 _38%_
**5.**	Rachel is the 15th tallest and the 15th shortest girl in her class. How many girls are in her class? **___**	29 _29%_	30 _34%_
**6.**	If 30 elves can wrap 30 gifts in 30 minutes, then 40 elves could wrap 40 gifts in ___ minutes.	30 _36%_	40 _59%_
**7.**	Jack can drink a bottle of cider in 6 days. Jill can drink a bottle of cider in 12 days. How long would it take them if they were drinking from the same bottle? ___ days	4 _31%_	9 _19%_
**8.**	In a basket of 60 apples that are either green or red, green apples are only 1/3 as common as red apples. How many apples are green? **___**	15 _14%_	20 _66%_

Note: Items are presented in descending order with respect to their biserial correlations with the other CRT items. MCE stands for most common error.

Participants next completed the MAT, whose eight items were drawn from the quantitative section of the Graduate Record Exam. (These eight items were also presented in random order on separate screens; see *SI Appendix*). Following these two tests, respondents encountered the items comprising our four DVs: beliefs (BEL), preferences regarding time and risk (PRF), our (nonmathematical) reflection scale (REF), and numeracy (NUM). The first three item blocks were presented in random order, as were the items *within* each block. (NUM, which was not preregistered, always appeared last.)

Our *Belief* scale (BEL) consisted of seventeen heterogeneous items grouped into four subscales, which measured the extent to which respondents endorsed the theory of evolution,^†^ but rejected religion (6–8), the paranormal (22), and “bullshit” (grammatical, but meaningless arrangements of emotionally evocative words) (10). The four subscales were presented in random order as were the items *within* each. The overall score was the average *z* score of the four subscales.^‡^

Our *Preference* scale (PRF) used nine items—three choices between a smaller-sooner reward and a larger-later reward and six choices between sure gains (or losses) and gambles offering the promise of a larger gain (or threat of a larger loss) (1). The score for this “scale” was somewhat atheoretic; we merely counted how often respondents chose the option associated with higher CRT scores in prior research (1)—the larger-later options for intertemporal choices, the riskier option in the domain of gains, and the safer options in the domain of losses.

Our *Reflection* scale (REF) consisted of eight items^§^ which were also intended to cue erroneous intuitions, but in nonmathematical contexts. The score was simply the number of items answered correctly, as per our preregistered answer key (Table 2).^¶^

**Table 2.** Reflection scale (REF)

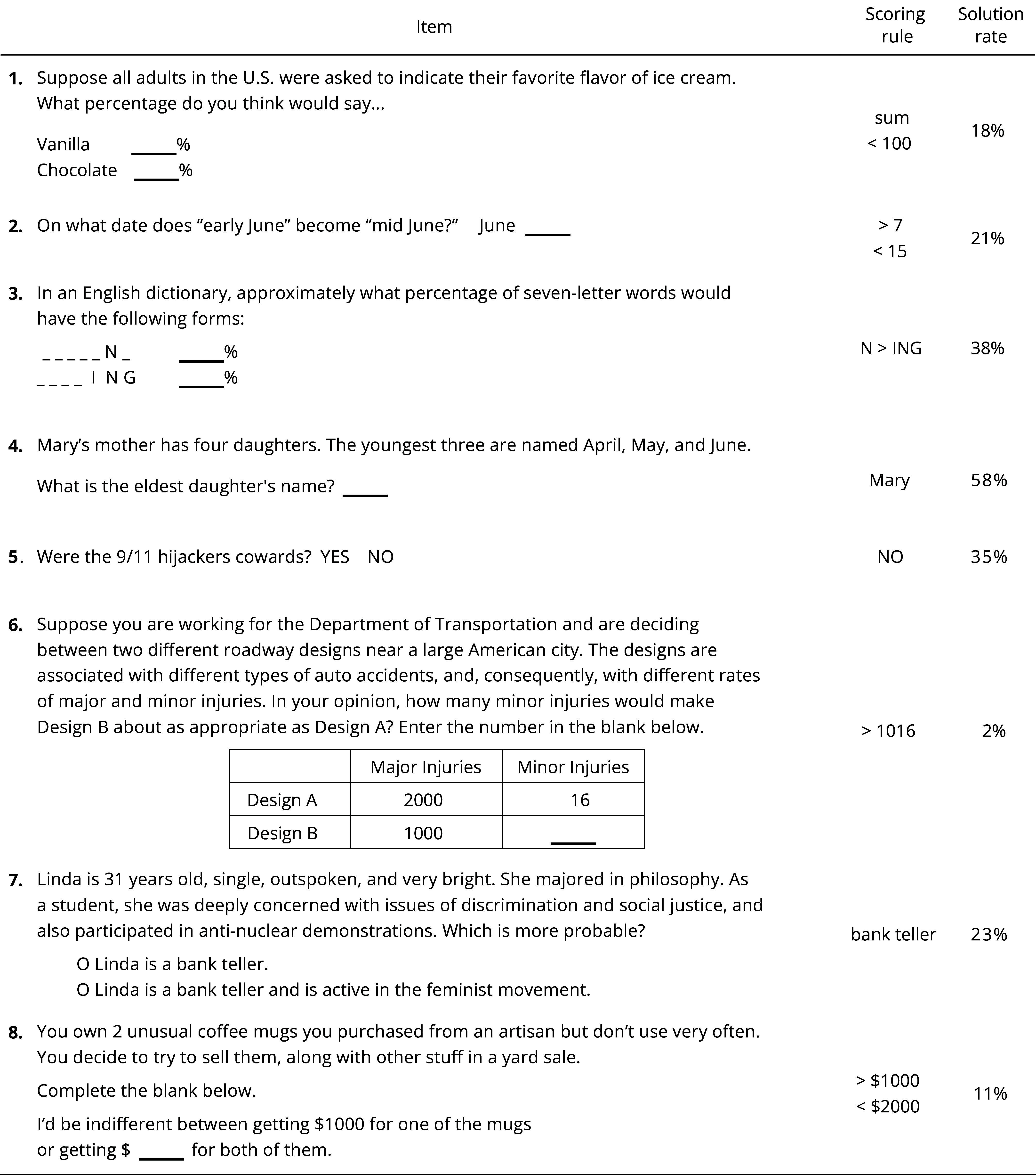

Note: Items are presented here in descending order with respect to their biserial correlations with the other REF items.

After answering our BEL, PRF, and REF items, participants learned which of the CRT and MAT items they had missed. Their incorrect initial responses were reproduced and they were given a chance to correct them. Our 8-item numeracy scale (NUM) appeared last. Its items only required arithmetic transformations of presented mathematical expressions.^#^ (Detailed descriptions of all scales and items are provided in *SI Appendix*.)

## Results

As noted earlier, our primary goal was to reexamine Attali & Bar-Hillel’s (14) claim that the CRT is nothing more than a math test (here, MAT). Unsurprisingly, we also found considerable correlation between the two tests (0.75 for observed scores, and 0.88 for latent or “true” scores). Nevertheless, confirmatory factor analysis revealed that a two-factor model described performance on those tests better than a one-factor model, reducing the RMS error of approximation by 22% (from 0.064 to 0.050; Satorra χ^2^(1) = 526, *P* < 0.001). Further details of this analysis are provided in our *SI Appendix*.

Table 3 shows the results of simultaneously regressing each of our four dependent variables onto CRT and MAT scores. The first specification in that table reveals substantial discriminant validity. Though the CRT was a significantly poorer predictor of Numeracy (20% less variance explained; *t* = 9.4), it was a significantly better predictor of everything else, explaining 16% more variance for BEL (*t* = 3.0), 30% more variance for PRF (*t* = 3.6) and 38% more for REF (*t* = 9.3). Additional details are provided in *SI Appendix*.

**Table 3.** DVs simultaneously regressed on CRT and MAT

		NUM	BEL	PRF	REF
**Specification 1:**	Observed MAT	**0.51**_0.02_	**0.17**_0.02_	**0.09**_0.02_	**0.12**_0.02_
	Observed CRT	**0.22**_0.02_	**0.29**_0.02_	**0.23**_0.02_	**0.46**_0.02_
**Specification 2:**	Latent MAT	**0.67**_0.02_	**0.21**_0.03_	**0.11**_0.03_	**0.17**_0.02_
	Observed CRT	**0.06**_0.02_	**0.24**_0.03_	**0.21**_0.03_	**0.42**_0.02_
**Specification 3:**	Observed MAT	**0.41**_0.02_	0.04 _0.03_	−0.03 _0.03_	−**0.06**_0.03_
	Latent CRT	**0.33**_0.02_	**0.42**_0.03_	**0.35**_0.03_	**0.65**_0.03_
**Specification 4:**	Latent MAT	**0.62**_0.04_	0.04 _0.04_	−0.05 _0.05_	−**0.09**_0.04_
	Latent CRT	**0.12**_0.04_	**0.42**_0.04_	**0.37**_0.05_	**0.67**_0.04_

Note: Numbers are standardized regression coefficients, bolded when significant (*P* < 0.05). Subscripts are the SE of these coefficients.

The CRT’s superior predictive validity suggests that it would add predictive validity to even a perfectly reliable version of MAT. To test this, we repeated the aforementioned regressions, but replaced *observed* MAT scores with *latent* MAT scores, which estimate the predictive validity of an infinitely long version of that test.^||^ Disattenuating only MAT is, of course, *conservative* with respect to claims regarding incremental predictive validity of the CRT.

As is all but guaranteed, using *latent* MAT scores increases MAT’spredictive validity (see specification 2 in Table 3). Nonetheless, *observed* CRT scores still yielded significant incremental predictive validity for BEL (*z* = 9.4), PRF (*z* = 7.7), and REF (*z* = 17.7). Moreover, these results remain statistically significant for 30 of the 34 individual *items* comprising those scales. (See *SI Appendix* for more details, including separate analyses for various demographic groups.)

If we use latent *CRT* scores (see specification 3 & 4 in Table 3), MAT remains the better predictor of NUM, but yields *no* appreciable incremental predictive validity for anything else (indeed, MAT actually becomes a significant *suppressor* variable for predicting REF). Thus, the CRT does appear to be measuring something beyond mathematical aptitude.^**^

## CRT as a Measure of Reflection

The results above, which show CRT to be a much stronger predictor of REF, coupled with the strong zero-order correlation between them (*r_CRTxREF_* = 0.88 after disattenuation), suggest that the component of the CRT which yields this incremental validity could also be measured by scales such as REF, which retain strong lures, but minimize math. This section examines how MAT and REF jointly contribute to explaining the predictive validity of the CRT.

If mathematical ability is operationalized via MAT scores and “reflection” is operationalized via REF scores, structural equation models can be used to assess how each contributes to the CRT’s relation with the remaining DVs. Unlike our foregoing analyses, in which REF functioned as a *dependent* variable (along with NUM, BEL, PRF), here it functions as an additional *independent* variable (along with MAT & CRT). This follows from our assumption that by stripping away mathematical content, our REF scale would help isolate cognitive reflection, as its items do not typically require (or, sometimes, even *permit*) the execution of numeric operations.

**Fig. 1.**
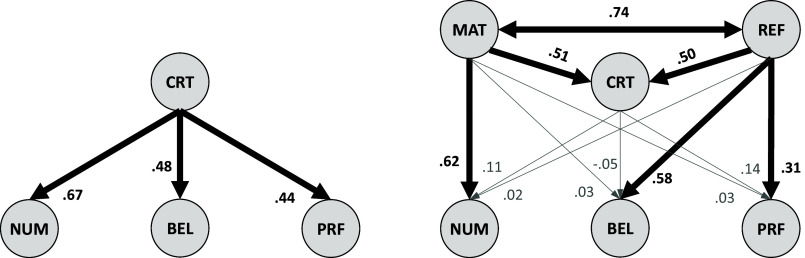
A decomposition of CRT’s relation with three DVs. Note: all variables are latent. Path coefficients are standardized (and bolded if *P* < 0.05).

The *Left* panel of Fig. 1 shows the CRT’s zeroth-order correlations with our focal DVs (*z*s = 34.1, 26.2, and 19.4). The expanded model in the *Right* panel reveals that those correlations can be fully explained by MAT and REF, leaving no residual predictive validity for the CRT itself.^††^ In the expanded model, MAT becomes the only significant predictor of NUM (z = 14.3), whereas REF becomes the only significant predictor of BEL (z = 5.8) and PRF (z = 3.1).

## Assessing the Role of Intuitive Lures

The preceding analysis suggests that the CRT’s incremental validity (with respect to various beliefs and preferences) is accounted for by its measurement of respondent’s reflective tendencies. We examine this further using our dual response paradigm, in which the opportunity to correct initial errors should reduce the importance of variation in the tendency to reflect upon initial intuitions (which becomes irrelevant if all participants are *told* when they are wrong).

On their first exposure to our 8-item CRT, respondents average 2.7 items correct. After being alerted to their incorrect responses and receiving a “second chance” to revise them, scores climbed to 3.9.^‡‡^ Examining relations with both initial scores and second chance scores helps dissociate respondents’ susceptibility to intuitive lures from their mathematical abilities, per se, since initial errors could include careless mistakes, whereas persistent errors more clearly reveal inadequate mathematical aptitude. Expressed differently, the second chance CRT should function more like an ordinary math test (5), because *telling* respondents which items they missed removes the need to spontaneously identify erroneous intuitions but preserves the mathematical requirements of the problems.

Table 4 confirms our prediction. Providing respondents a chance to correct their initial, impulsive errors made the CRT a better predictor of NUM and MAT but a worse predictor of PRF (time and risk preferences) and REF (reflection in nonmathematical contexts). Overall, the CRT’s relation with BEL was not affected by the chance to correct initial impulsive errors, though this null effect at the scale level masked heterogeneous effects across the various *items* of that scale. (We present the item-level results in our *SI Appendix*.)

**Table 4.** DVs simultaneously regressed on CRT and second chance CRT

	NUM	MAT	BEL	PRF	REF
CRT	**0.11**_0.03_	**0.33**_0.02_	**0.21**_0.03_	**0.28**_0.03_	**0.48**_0.03_
2nd chance CRT	**0.55**_0.03_	**0.47**_0.02_	**0.23**_0.03_	0.02 _0.03_	**0.08**_0.03_
Effect of 2nd chance on validity	**+0.44**_0.05_	**+0.14**_0.04_	+0.02 _0.06_	−**0.26**_0.06_	−**0.40**_0.05_
t-statistic	**8.9**	**3.3**	0.3	−**4.1**	−**7.3**

Note: Second chance CRT score includes items solved upon first exposure and those successfully corrected after initial errors were revealed. Numbers in the first two rows are standardized regression coefficients. Numbers in the third row are differences between the first and second row. Subscripts are SE. Bolded values indicate statistical significance (*P* < 0.05).

## Conclusions

In contrast to recent claims (14–20), the CRT is *not* just a math test. Though it may be legitimately used *as such*, it yields surplus predictive validity in many domains, because it measures *cognitive reflection*—a construct which researchers have typically attempted to assess using *tricky* math items, but *math* items nonetheless.

Here, we attempted to isolate the reflection component of the CRT with our REF Scale (eight items intended to measure reflection, without requiring much or any math). Though we consider it an improvement over prior attempts (24, 25), we acknowledge that our strongest claims rely heavily on disattenuation. That said, our “prophetic” analyses using *latent* REF scores imply that the reflection component of the CRT may be sufficient to explain its surplus predictive validity relative to more traditional math tests, and we assume that if it could be made more reliable (e.g., by lengthening it), it *would* measure cognitive reflection separately from mathematical ability.

Our dual response paradigm supports this interpretation, by distinguishing participants’ ability to *spontaneously* suppress erroneous intuitions from their ability to “do math.” Giving respondents a chance to correct their initial errors obviously reduces the relevance of differences in the ability to *spontaneously* suppress erroneous intuitions, and, as predicted, made the CRT function more like other math tests, increasing its correlation with our mathematical scales (NUM & MAT), but reducing its correlation with our other DVs. In summary, our data suggest that the CRT measures cognitive reflection, that its surplus predictive validity can be explained in terms of this construct, and that this construct can be measured by items not involving math.

## Supplementary Material

Appendix 01 (PDF)

## Data Availability

.csv data have been deposited in osf (DOI: 10.17605/OSF.IO/9J8MC) (34).

## Footnotes

^*^Our eight MAT items formed a slightly more reliable scale (Cronbach’s α = 0.87; ω_total_ = 0.90) than our eight CRT items (Cronbach’s α = 0.84; ω_total_ = 0.86).

^†^As designed by Shane Frederick.

^‡^Most of these items were chosen because past research had found correlations with the CRT. However, we added four inspirational quotes to the profundity subscale (see items 1 to 4 in *SI Appendix*, Table S6). For these items, we were unsure whether those scoring higher on the CRT would view them as more or less profound. Accordingly, our preregistered decision was to evaluate the *difference* in judged profundity between the four inspirational quotes and the four bullshit phrases—we predicted that greater reflection would yield a stronger repudiation of bullshit than banality. Though we decided to abide by this for the results presented in the main text, it is not the *only* way to score our BEL scale. We could, instead, have simply omitted the four inspirational quotes from the analysis or scored them as we did the bullshit items. Either decision would have increased discriminant validity between CRT and MAT, as discussed in *SI Appendix*, where we present item-level analyses of this scale.

^§^Credit for items: #1 Shane Frederick, Ryan Hauser & Asher Lawson; #2 Amos Tversky & Daniel Kahneman (23); #3 Ryan Hauser; #4 was part of the “CRT-2” (24) and “Verbal CRT” (25); #5 Shane Frederick; #6 Shane Frederick & Eldar Shafir. #7 Daniel Kahneman and Amos Tversky (26); #8 Shane Frederick.

^¶^In one of the four survey platforms (*Prolific*), a coding error prevented responses to REF items #6 & #8 from being recorded. Thus, for *this* part of the data, we replaced the missing data (0s or 1s) with the solution rates across the other three samples (0.023 for #6 and 0.106 for #8). This is conservative with respect to all of our main results. Those two items had predictive validity elsewhere, and, thus, assigning an identical value to all Prolific respondents nullifies their predictive value for that subsample (and dilutes their predictive value for the full sample). Moreover, all results replicate if we simply exclude those two items from the REF scale everywhere. See *SI Appendix* for further details.

^#^Shane Frederick developed this scale, surmising that it would isolate the putative construct of Numeracy, as we construe it, better than other popular Numeracy scales (27–29).

^||^We estimate scores on underlying latent variables using R’s Lavaan library, with item weights and variances allowed to vary freely, as pre-registered (30). This is an improved version of disattenuation procedures used in the past (31–33). If item weights and variances are fixed, latent variable techniques converge with older dissattenuation procedures.

^**^We acknowledge that many of the items used in our BEL and PRF “scales” were *selected* because they had been shown to correlate with variants of three items we use in our CRT, whereas none of the MAT items benefitted from any such selection.

^††^Though our SEM results support our interpretation of REF as a “purer” measure of cognitive reflection, its low reliability (Cronbach’s α = 0.47; ω _total_ = 0.57) means it is not yet an especially *good* measure—in part, because two of its eight items were missing for a fourth of our sample, and in part because those two items were so difficult that they could not, in any case, contribute much to its reliability (and validity). Thus, given the low reliability of our REF scale, model results diagrammed above rely on disattenuation.

^‡‡^Though we required participants to re-enter *an* answer, we did not prevent them from re-entering their original answer. Nonetheless, among the 4,180 respondents who initially made one or more errors, 71% successfully corrected at least one of them. See *SI Appendix* for further discussion.
