# Supplementary material for: Cognitive reflection is a distinct and measurable trait
Source: Proc Natl Acad Sci U S A. 2024 Nov 27;121(49):e2409191121. doi: 10.1073/pnas.2409191121 (PMC11626181; doi:10.1073/pnas.2409191121)
Supplement: Supplementary file 1 — Appendix 01 (PDF) [file pnas.2409191121.sapp.pdf]

Supplementary Information

Contents

1. Sample construction and composition……………………………………….…..……...……2

2. Detailed descriptions of our NUM, MAT, BEL & PRF scales……...……..……..…………......5

3. Factor Analysis of CRT and MAT items….………………………………………...……9

4. Predictive validities of CRT and MAT… ..……13

5. Incremental predictive validity of CRT for individual items …..……15

6. Scale average by demographic group…….……….………..……….………...……………………17

7. Incremental predictive validity of CRT by demographic group…………………………..…..19

8. Effect of 2nd chance on predictive validity for individual items……..………..……………...…22

9. Results using a 6-item Reflection Scale…………………….……………...………….………25

10. Results with alternative scoring of the profundity subscale …………………...…………..…27

11. Supplementary references…………………………………………………………………....29

1. Sample construction and composition

As pre-registered (https://doi.org/10.17605/OSF.IO/NXKE3), we recruited almost 9000 participants from four online survey platforms (mTurk, Qualtrics, Forthright, & Prolific). Those who passed all eight screener questions (see Table S1, below) could stop at this point and receive $1, or elect to continue to a longer survey for an additional $10. The longer survey was described as taking "about 45 minutes" (the actual median time to complete the entire survey was 37 minutes, which includes the screeners and some items not mentioned in this paper).

Table S1: questions from screening block and passing rates

Screener question	Response options	Acceptable Answer	% passing Screener
1. Informed consent	Yes, No	Yes	99%
2. Are you a Native Speaker of English?	Yes, No	Yes	98%
3. Have you ever been bitten by an insect?	Yes, No	Either[1]	100%
4. Are you a human?	Yes, No	Yes	98%
5. Have you ever been bitten by a great white shark?	Yes, No	No	92%
6. Have you ever suffered a fatal heart attack?	Yes, No	No	92%
7. In which of these grades are students about 5 years old?	Kindergarten, third grade, fifth grade, seventh grade, ninth grade	Kindergarten	89%
8. Which of these is NOT associated with Halloween?	Trick-or-treating, Pumpkins, Costumes, Ghosts, Eating turkey	Eating turkey	86%
9. Which of these is an American zip code?	14 Monroe Avenue, 112, 789, 02318, 7TX 4LZ	02318	93%

[1] We debated whether those who claimed to have never been bitten by an insect were responding honestly or were being inattentive or perverse. Though we ultimately chose *not* to screen on this item, we retained it in part to "mask" the purpose of our other binding screeners and in part to slake our curiosity regarding it. Among the 4407 who passed all the other screeners and our embedded attention check, 342 (8%) claimed to have never been bitten by an insect. That subset scored lower on every measure of cognitive ability.

Among the 6,894 participants who passed all screener questions, 4,628 chose to participate in the longer survey. However, we pre-registered one further "embedded" attention check placed *within* the Preferences block of the main survey, and excluded 221 more respondents for choosing a 30% chance of $100 over $200 for sure. This left 4,407 respondents for our analyses. Sample sizes at each stage are reported in Table S2, for each survey platform.

Table S2: sample sizes for each stage and survey platform

	Total	mTurk	Qualtrics[2]	Forthright	Prolific
Started	8990	2883	2104	1889	2104
Passed screener	6894	1423	1919	1581	1971
Began main survey	4628	1126	1207	1193	1102
Final N*	4407	1028	1141	1147	1091

*** *those who finished survey and passed an additional "embedded" attention check*

Our final sample is moderately diverse, with ages ranging from under 20 to over 90, education levels ranging from some high school to doctoral degrees, and incomes ranging from under $20,000 per year to over $300,000. Men and women were, roughly, equally represented, and about one fourth of our participants were minorities. Full demographic details are presented in Table S3.

[2] We later discovered that 34 participants from our Qualtrics sample were not "screened out" despite providing one or more inadmissable responses for screener item(s) 2, 4, or 5. We retained these people in the analyzed sample. That decision has no substantial effect on any of our results.

Table S3: demographics of final sample

**Sex**	Female: **50.6%**<br>Male: **48.5%**<br>Other: **0.8%**<br>No Response: **0.0%**
**Age**	Under 20: **0.8%**<br>20 to 29: **16.1%**<br>30 to 39: **25.8%**<br>40 to 49: **18.6%**<br>50 to 59: **14.9%**<br>60 to 69: **14.8%**<br>70 to 79: **7.7%**<br>Over 80: **1.2%**<br>No Response: **0.1%**
**Race**	White: **75.7%**<br>Black: **8.3%**<br>Hispanic: **4.6%**<br>Asian: **4.4%**<br>American Indian: **0.7%**<br>Pacific Islander: **0.1%**<br>Middle Eastern: **0.0%**<br>Other: **0.9%**<br>Selected Multiple Races: **5.2%**<br>No Response: **0.1%**
**Political Party**	Democrat: **46.9%**<br>Republican: **25.8%**<br>Neither: **25.1%**<br>Prefer not to answer: **2.2%**<br>No Response: **0.0%**

**Income**	< $20,000: **11.3%**<br>$20,000 to $39,999: **20.1%**<br>$40,000 to $59,999: **20.8%**<br>$60,000 to $79,999: **16.2%**<br>$80,000 to $99,999: **10.4%**<br>$100,000 to $119,999: **6.6%**<br>$120,000 to $139,999: **3.9%**<br>$140,000 to $159,999: **3.6%**<br>$160,000 to $179,999: **1.9%**<br>$180,000 to $199,999: **1.4%**<br>$200,000 to $219,999: **0.8%**<br>$220,000 to $239,999: **0.6%**<br>$240,000 to $259,999: **0.7%**<br>$260,000 to $279,999: **0.2%**<br>$280,000 to $299,999: **0.3%**<br>More than $300,000: **0.7%**<br>No Response: **0.4%**
**Education**	Some high school: **1.3%**<br>High school degree: **16.2%**<br>Some college: **30.0%**<br>Bachelor’s degree: **37.1%**<br>Master’s degree: **13.5%**<br>Doctoral degree: **1.8%**<br>No Response: **0.0%**

2. Detailed descriptions of our NUM, MAT, BEL, & PRF scales

Our CRT and REF scales are fully described in the main text. Our eight-item numeracy test (NUM), is shown below in Table S4. As with the CRT and REF scales, these eight items were presented together as a block, though in random order on separate screens.

Table S4: our 8-item Numeracy test (NUM)

Question	Solution Rate	MCE Rate
1. Express 1/20 as a decimal ____	0.05 64%	0.2 10%
2. Express 3/5 as a decimal ____	0.60 71%	3.5 6%
3. Express 11/4 as a decimal ____	2.75 64%	11.4 5%
4. 0.875 is the same as ____/ 8	7 67%	1 8%
5. 1.25 is the same as ____/ 4	5 68%	1 14%
6. 0.0375 is the same as 3 / ____	80 29%	8 16%
7. What is 10/3 ÷ 5/9 ____	6 43%	0.6 4%
8. Two is ____% as big as five	40 38%	2.5 9%

Note: *Items are presented in descending order with respect to their biserial correlations with the other NUM items. MCE stands for Most Common Error.*

Our Mathematical Aptitude Test (MAT) is shown below in Table S5. It consisted of eight items drawn from the quantitative section of the Graduate Record Exam which we anticipated (correctly) would lack a salient intuitive lure (the 2nd subscripts reveal that, unlike the CRT, the various errors were much less “concentrated.”) These items were also presented in random order on separate screens, and the score was simply the number correct.

Table S5: an 8-item Mathematical Aptitude Test (MAT)

Item	Solution Rate	MCE Rate
**1.** What is the area of the grey region? ____ 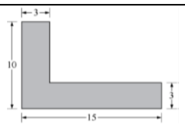	66 37%	75 9%
**2.** If the average (arithmetic mean) of 6, 8, and x is 6, then x = ____	4 44%	6 16%
**3.** In the figure, if x = 2y, then y = ____ 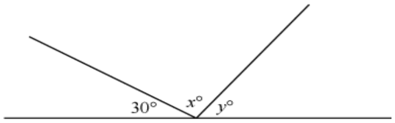	50 29%	15 11%
**4.** The three sides of the triangle are equal and the area of the square region is 100. What is the perimeter of the triangle? ____ 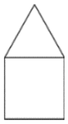	30 32%	75 13%
**5.** If 9 is 3/4 of n, what number is 5/6 of n? ____	10 50%	12 8%
**6.** If a square has the same area as a rectangle that is 16 by 4, then a side of the square has what length? ____	8 44%	4 23%
**7.** One wire has a diameter of 0.005 mm. Another wire has a diameter of 0.02 mm. The thicker wire is ____times thicker.	4 42%	0.02 6%
**8.** A wall that is 12 feet wide and 10 feet high is to be painted. A blackboard that is 5 feet wide and 3 feet high is affixed to the wall and is the only portion of the wall that will not be painted. The area of the portion of the wall that will be painted is how many square feet? ____	105 53%	15 8%

Note: *Items are presented in descending order with respect to their biserial correlations with the other MAT items. MCE stands for Most Common Error.*

The mean scores on our four 8-item tests (NUM, MAT, CRT, and REF) were 4.4, 3.3, 2.7, and 2.1, with standard deviations of 2.7, 2.8, 2.5, and 1.5, respectively.

Our *Belief* scale (BEL) is shown in Table S6. It consisted of four subscales, which were presented in random order, as were the items within each. Our pre-registered measure of *Reflective Beliefs* consisted of summing item scores within each subscale, normalizing them, and then taking the mean of those four z-values.

Table S6: Belief Scale (BEL)

**Profundity**	Mean
1. All endings are also beginnings. We just don't know it at the time.	3.82
2. The best way to predict your future is to create it.	3.75
3. You miss 100% of the shots you don't take.	3.56
4. A wet person does not fear the rain.	3.24
5. Your heart requires the flow of actions.	3.10
6. Hidden meaning transforms unparalleled abstract beauty.	3.06
7. Good health imparts reality to subtle creativity.	2.92
8. Wholeness quiets infinite phenomena.	2.82
Not at all Profound 1 2 3 4 5 Very Profound	
**The Paranormal**	
1. During altered states, such as sleep or trances, the spirit can leave the body.	2.41
2. The Loch Ness monster of Scotland exists.	2.13
3. If you break a mirror, you will have bad luck.	1.85
Strongly disagree 1 2 3 4 5 Strongly agree	
**Evolution**[3]	
1. Evolution is just a theory, and more like a hypothesis than a fact.	2.62
2. Many structures of the human body, such as the eye, are too complex to have arisen from evolution alone.	2.77
3. Whales are more closely related to sharks than to dogs.	3.44
Strongly disagree 1 2 3 4 5 Strongly agree	
**Religiosity**[4]	
1. Do you believe in God? Yes (3) No (1) Not Sure (2)	2.48
2. How religious are you? Not at all 1 2 3 4 5 Extremely	2.66
3. How often do you pray? Never (1)… Multiple times a day (9)	5.13

Our *Preferences* scale (PRF) is shown in Table S7. Items were presented in random order on separate screens and, unlike the table, the position of response options (left or right) was randomized. Here, the overall score was simply number of times respondents chose Option "B" –

[3] These three items were developed by Shane Frederick.

[4] Item #3 had nine response options which were seriated vertically. The seven intermediate options (not shown above) were: about once a year (2), a few times a year (3), about once a month (4), more than once a month (5), about once a week (6), more than once a week (7), once a day (8)

the preference which diagnosed higher CRT scores in the data from Frederick (2005): the larger, later options for the three intertemporal choices (items #1, #2, & #3), the risky options in the domain of gains (items #4, #5, #6, & #7), and the safer (and higher EV) options in the domain of losses (items #8 & #9).[5]

Table S7: Preferences Scale (PRF)

Option A	Option B	% Option B
1. $3,400 this month	$3,800 next month	63%
2. $100 now	$140 next year	27%
3. $100 now	$1,100 in 10 years	28%
4. Gain $60 for sure	1% chance to gain $5,000	18%
5. Gain $100 for sure	3% chance to gain $7,000	22%
6. Gain $500 for sure	15% chance to gain $1,000,000	44%
7. Gain $100 for sure	75% chance to gain $200	49%
8. 3% chance to lose $7,000	lose $100 for sure	56%
9. 75% chance to lose $200	lose $100 for sure	72%

[5] Here our classification of the "reflective" choice were based on results presented by Frederick (2005). Our pre-registration miscoded #8 and #9. We coded Item #4 (where the riskier option had lower EV) as intended, but note that this decision was motivated by (weak) evidence from Frederick (2005) that the greater risk tolerance exhibited by more reflective respondents may even yield more risk *seeking* behavior (provided the penalty in expected value was sufficiently modest and/or the sure gain sufficiently small). We find no relation (a "null" result which is nevertheless informative, because it indicates that the stronger relations are not "just" greater reliance on expected value among those most capable of performing those computations.)

3. Factor Analysis of CRT and MAT items

Figure S1 presents item loadings for the one-factor and two-factor models of the CRT and MAT items. As reported in the main text, the two-factor solution significantly improves model fit relative to the one factor solution (Satorra $\chi^2(1) = 526$, $p < 0.001$). The one factor solution has an RMSEA of 0.064 on 104 degrees of freedom (90% CI: .061 to .066), CFI of 0.933, and TLI of 0.922. The two-factor solution has an RMSEA of 0.050 on 103 degrees of freedom (90% CI: .047 to .052), CFI of 0.959, and TLI of 0.953.

Figure S1: confirmatory factor analysis of CRT and MAT items

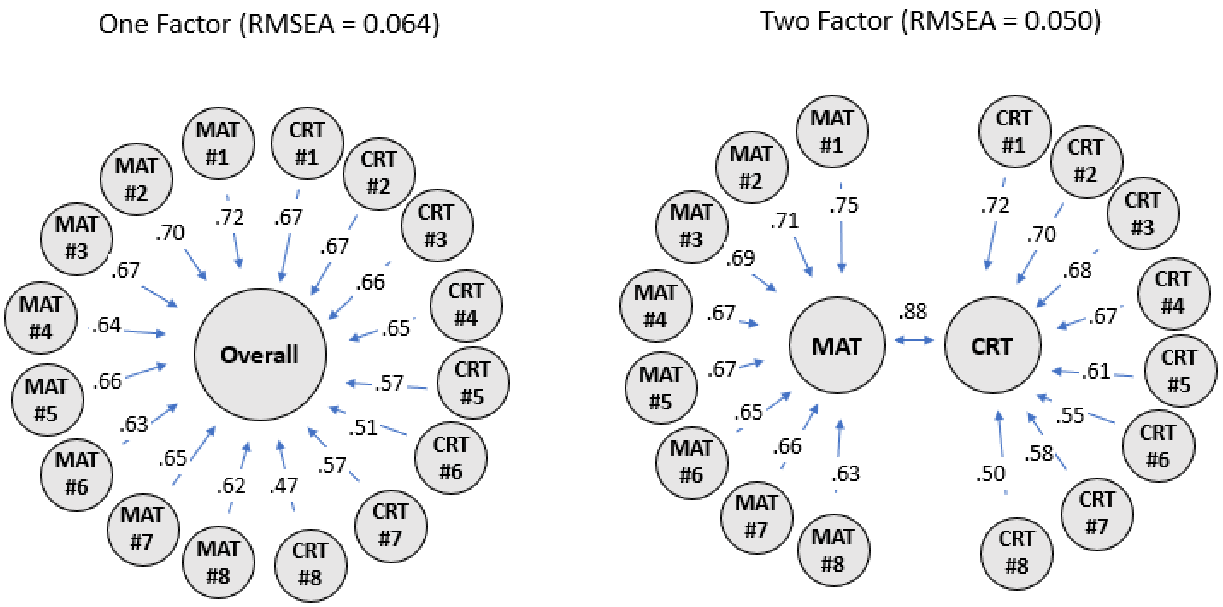

***Note**: all factor loadings are standardized. Item numbering refers to Table 1 and Table S5.*

Table S8 presents an exploratory factor analysis of the CRT and MAT with a promax rotation that allows for two correlated factors. The two factors are correlated -0.79, and perfectly separate the CRT items from the MAT items. All 8 MAT items load more strongly on the first

factor than on the second, while all 8 CRT items load more positively on the second factor than on the first.

Table S8: exploratory factor analysis of CRT and MAT items with promax rotation

		Factor 1	Factor 2
**CRT**	1. Drill & hammer	-.06	**.80**
	2. Dog & cat	.13	**.59**
	3. Baby bird	.24	**.46**
	4. Toaster price	.30	**.39**
	5. Rachel	-.02	**.63**
	6. Elves	-.12	**.68**
	7. Jack & Jill	.22	**.38**
	8. Apples	.01	**.51**
**MAT**	1. Area of grey region	**.79**	-.03
	2. Mean of 6, 8, & x = 6	**.53**	.21
	3. Angle of y	**.60**	.10
	4. Triangle perimeter	**.78**	-.11
	5. 5/6 of n	**.59**	.09
	6. Side of square	**.66**	.00
	7. Diameter of wire	**.46**	.22
	8. Painted wall	**.57**	.07
Proportion of Variance explained		.21	.17

***Note**: Factor loadings are regression based, with the larger of the two factor loadings bolded.*

Table S9 presents an exploratory factor analysis of the CRT and MAT with a varimax rotation that requires factors to be uncorrelated. Again, the two factors perfectly separate the two sets of items. Although all items load substantially on both factors (as we would expect with orthogonal factors and a high correlation between observed scores), all MAT items load more heavily on the first factor than on the second, while all CRT items load more heavily on the second factor than on the first. Further, the distributions of factor loadings do not overlap. All MAT items have higher loading on factor 1 than any CRT item, and all CRT items have higher loading on factor 2 than any MAT item.

Table S9: exploratory factor analysis of CRT and MAT items with varimax rotation

		Factor 1	Factor 2
**CRT**	1. Drill & hammer	.31	**.69**
	2. Dog & cat	.39	**.58**
	3. Baby bird	.43	**.51**
	4. Toaster price	.45	**.47**
	5. Rachel	.28	**.56**
	6. Elves	.20	**.55**
	7. Jack & Jill	.37	**.43**
	8. Apples	.24	**.45**
**MAT**	1. Area of grey region	**.70**	.31
	2. Mean of 6, 8, & x = 6	**.57**	.41
	3. Angle of y	**.59**	.34
	4. Triangle perimeter	**.66**	.24
	5. 5/6 of n	**.58**	.33
	6. Side of square	**.60**	.28
	7. Diameter of wire	**.52**	.39
	8. Painted wall	**.55**	.31
Proportion of Variance explained		.21	.24

***Note**: factor loadings are regression based, with the larger of the two factor loadings bolded.*

Figure S2 presents a zero-order correlation matrix for the CRT and MAT items. Its upper triangle are point-biserial correlations and its lower triangle are tetrachoric correlations. The figure confirms the factor analyses by showing that correlations within type of item are higher than correlation between types of items (point-biserial: mean $r_{CRTxMAT} = 0.37$ vs. mean $r_{CRTxCRT} = 0.39$ & mean $r_{MATxMAT} = 0.46$; tetrachoric: mean $r_{CRTxMAT} = 0.58$ vs. mean $r_{CRTxCRT} = 0.62$ & mean $r_{MATxMAT} = 0.68$).

Figure S2: correlation matrix of CRT and MAT items

		CRT								MAT							
		#1	#2	#3	#4	#5	#6	#7	#8	#1	#2	#3	#4	#5	#6	#7	#8
CRT	#1		.53	.45	.46	.47	.44	.39	.44	.43	.45	.44	.40	.39	.37	.43	.36
	#2	.79		.50	.47	.40	.38	.38	.32	.44	.49	.42	.38	.43	.39	.44	.39
	#3	.76	.73		.46	.39	.42	.39	.25	.44	.48	.38	.36	.45	.41	.44	.45
	#4	.70	.68	.70		.39	.31	.35	.33	.45	.47	.41	.40	.44	.39	.43	.40
	#5	.69	.62	.64	.59		.39	.38	.33	.37	.38	.36	.31	.34	.34	.35	.32
	#6	.67	.56	.64	.48	.59		.33	.29	.32	.31	.31	.25	.28	.30	.30	.30
	#7	.60	.58	.62	.54	.58	.51		.31	.39	.37	.37	.35	.35	.35	.37	.35
	#8	.72	.59	.51	.59	.57	.52	.55		.33	.30	.34	.31	.26	.27	.28	.21
MAT	#1	.66	.65	.67	.66	.57	.50	.59	.58		.51	.54	.55	.47	.50	.47	.50
	#2	.71	.69	.70	.68	.59	.47	.58	.56	.72		.47	.46	.51	.43	.50	.44
	#3	.67	.63	.61	.62	.56	.49	.57	.59	.77	.71		.50	.44	.45	.44	.39
	#4	.61	.57	.58	.60	.48	.40	.55	.54	.77	.68	.73		.43	.50	.41	.39
	#5	.64	.64	.65	.65	.54	.43	.55	.51	.70	.72	.69	.66		.43	.46	.46
	#6	.59	.57	.61	.58	.53	.47	.55	.52	.72	.63	.68	.73	.63		.40	.41
	#7	.67	.64	.65	.64	.54	.47	.56	.52	.68	.71	.66	.61	.66	.58		.42
	#8	.60	.58	.65	.61	.53	.47	.56	.42	.74	.64	.62	.61	.66	.60	.62	

***Note**: Upper triangle are point-biserial correlations. Lower triangle are tetrachoric correlations. Item numbering refers to Table 1 and Table S5.*

4. Predictive validities of CRT and MAT

Table S10 presents a zero-order correlation matrix, with correlations between *observed* scores reported in the upper-right triangle, correlations between *latent* scores reported in the lower-left triangle (based on a confirmatory factor analysis with item weights and variances allowed to vary freely), and measures of *scale reliability* (ω_{total}) reported along the diagonal.

Table S10: zero-order correlations

	NUM	MAT	CRT	BEL	PRF	REF
NUM	**.87**	.67	.60	.29	.20	.38
MAT	*.73*	**.90**	.75	.38	.26	.47
CRT	*.67*	*.88*	**.86**	.41	.29	.55
BEL	*.30*	*.41*	*.48*	**.89**	.26	.34
PRF	*.24*	*.37*	*.43*	*.33*	**.68**	.23
REF	*.56*	*.74*	*.88*	*.55*	*.44*	**.57**

Note: *Correlations between observed scores are in the upper-right triangle. Correlations between latent scores are in italics in the lower-left triangle. The* ***bold*** *numbers along the diagonal are* ω_{total}.

Non-parametric correlations (Spearman's ρ) replicate the differences in predictive validity between observed scores on CRT and MAT. MAT continues to outperform CRT as a predictor of Numeracy (t = 8.8), while CRT continues to outperform MAT as a predictor of Beliefs (t = 2.6), Preferences (t = 2.4), and Reflection Scale scores (t = 7.8).

Table S11 presents a commonality analysis. It decomposes the predictive validity of observed scores on the CRT and MAT into three components: the predictive validity from CRT's unique variance, the predictive validity from MAT's unique variance, and the predictive validity from their shared variance. It shows that their shared variance always has the most explanatory power, but the MAT's unique variance has more explanatory power than the CRT's unique

variance for NUM, while the CRT's unique variance has more explanatory power than the MAT's unique variance for the other three DVs.

Table S11: commonality analysis

	NUM	BEL	PRF	REF
Variance common to CRT and MAT	34.2	13.5	6.4	21.3
Variance unique to CRT	2.1	3.5	2.3	9.1
Variance unique to MAT	11.3	1.2	0.3	0.7
R^2 for DV ~ CRT + MAT	47.5	18.2	9.0	31.1

Note*: The column labels are DVs. The row labels are components of CRT and MAT variance. The numbers of are percentages of variance in each DV that can be explained by each component of CRT and MAT variance.*

5. Incremental predictive validity of CRT for individual items

For item level analyses, we regressed responses to each of our 34 items individually onto *latent* MAT scores and *observed* CRT scores simultaneously (see Table S12). The numbers in the table are the standardized regression coefficients and represent the expected change (in standard deviations) in the response to that item given one standard deviation change in scores on the CRT or MAT (holding the score of the other test constant). Statistically significant ($p < .05$) coefficients are bolded.

The table reveals that CRT confers significant incremental predictive validity for 30 of our 34 items. The only four exceptions were BEL item #4 (profundity judgments of the inspirational quote "*A wet person does not fear the rain*.") PRF items #4 & #5 (whether they'd choose a 1% of $5000 over a sure $60, or a 3% of $7000 over a sure $100) and REF item # 5 (whether they'd characterize the 9/11 hi-jackers as cowards.) Note that for *this* table we scored all items the same way, with positive coefficients revealing a negative relation between performance (on the CRT and MAT) and judged profundity of the target statement, whether the statement was a cliché inspirational quote or gibberish.

Table S12: regression of individual items on MAT and CRT simultaneously

		**MAT** (latent)	**CRT** (observed)
**BEL** Inspirational Quotes	1. Endings are beginnings	-.01	**.14**
	2. Create your future	-.00	**.15**
	3. Shots untaken	.04	**.08**
	4. Fear the rain	**.06**	.04
**BEL** Bullshit Phrases	5. Flow of actions	**.12**	**.22**
	6. Hidden meaning	**.08**	**.19**
	7. Imparts reality	**.07**	**.24**
	8. Infinite phenomena	**.10**	**.16**
**BEL** Evolution	1. It's just a theory	**.15**	**.12**
	2. Eye is too complex	**.12**	**.16**
	3. Whales are similar to sharks	**.20**	**.06**
**BEL** Paranormal	1. Spirit leaves body	**.16**	**.13**
	2. Loch Ness monster exists	**.13**	**.11**
	3. Broken mirrors = bad luck	**.17**	**.13**
**BEL** Religiosity	1. Believe in god	**.08**	**.21**
	2. How religious	**.07**	**.20**
	3. Prayer frequency	**.09**	**.20**
**PRF** Time	1. $3400 vs. $3800 in a month	**.13**	**.11**
	2. $100 vs. $140 in a year	**.13**	**.10**
	3. $100 vs. $1100 in ten years	**.06**	**.13**
**PRF** Risk (gains)	4. $60 vs. 1% $5000	-.02	-.02
	5. $100 vs. 3% $7000	-.01	.03
	6. $500 vs. 15% $1,000,000	**.07**	**.19**
	7. $100 vs. 75% $200	**.07**	**.12**
**PRF** Risk (losses)	8. 3% $7000 vs. $100	-.05	**.07**
	9. 75% $200 vs. $100	**.06**	**.10**
**REF**	1. Complementarity	**.10**	**.25**
	2. Middle of June begins	**.16**	**.18**
	3. _N_ vs. ING	**.17**	**.14**
	4. Mary's mother	.01	**.37**
	5. 9/11 Hijackers	**.19**	.05
	6. Minor injuries	**.09**	**.12**
	7. Linda	**-.11**	**.26**
	8. Mugs	.00	**.16**

Note: *numbers are standardized coefficients from the regression of each item onto latent MAT and observed CRT simultaneously. Standard errors are about 0.03 for each estimate. Bold coefficients are statistically significant (p < .05).*

6. Scale average by demographic group

Table S13 through S19 presents scale scores disaggregated by demographic group and survey platform. All scales are normalized to have mean 0 and standardized deviation 1, so differences between group means can be *roughly* interpreted as Cohen's *D*s.[6] Subscripts are standard errors.

Overall, men score higher than women. Asians scored highest, followed by Whites, followed by Hispanics and Blacks. Those with more education and higher incomes score higher. Younger participants scored higher. Independents score highest, followed by Democrats, followed by Republicans. Participants from *Prolific*'s survey platform scored highest, followed by Mturk, followed by Qualtric and Forthright.

Table S13: Scores split by **gender**

	N	NUM	MAT	CRT	BEL	PRF	REF
Female	2234	$-.17_{.02}$	$-.18_{.02}$	$-.22_{.02}$	$-.14_{.02}$	$-.14_{.02}$	$-.14_{.02}$
Male	2138	$.17_{.02}$	$.18_{.02}$	$.22_{.02}$	$.14_{.02}$	$.15_{.02}$	$.13_{.02}$
Other	35	$.39_{.16}$	$.44_{.17}$	$.30_{.15}$	$.85_{.13}$	$.22_{.15}$	$.55_{.13}$

Table S14: Scores split by **race / ethnicity**

	N	NUM	MAT	CRT	BEL	PRF	REF
White	3336	$.00_{.02}$	$.03_{.02}$	$.04_{.02}$	$.03_{.02}$	$.00_{.02}$	$.05_{.02}$
Black	365	$-.27_{.06}$	$-.43_{.04}$	$-.46_{.04}$	$-.35_{.05}$	$-.22_{.05}$	$-.34_{.05}$
Hispanic	204	$-.20_{.07}$	$-.32_{.07}$	$-.23_{.06}$	$-.08_{.06}$	$.05_{.06}$	$-.21_{.07}$
Asian	193	$.53_{.07}$	$.55_{.07}$	$.56_{.08}$	$.23_{.07}$	$.26_{.07}$	$.18_{.08}$
Other	309	$.12_{.05}$	$.03_{.06}$	$-.08_{.05}$	$-.03_{.05}$	$.06_{.06}$	$-.07_{.06}$

Note: *The "Other" category aggregates those who selected Middle Eastern, Pacific Islander, Other, multiple races, or who declined to respond.*

[6] Since the *total* variance is standardized to 1 in these tables, differences in the standardized scores underestimate the effect sizes (in terms of Cohen's *D*) because the pooled variance is smaller than the total variance for any two groups with different means.

Table S15: Scores split by **age**

	N	NUM	MAT	CRT	BEL	PRF	REF
Under 35	1454	$.20_{.03}$	$.10_{.03}$	$.12_{.03}$	$.00_{.03}$	$.03_{.03}$	$.12_{.03}$
35 to 55	1643	$-.01_{.02}$	$-.03_{.02}$	$.03_{.03}$	$.00_{.03}$	$.04_{.02}$	$.06_{.03}$
Over 55	1307	$-.21_{.03}$	$-.07_{.03}$	$-.18_{.03}$	$.01_{.02}$	$-.08_{.03}$	$-.21_{.03}$

Note: *This analysis omits 3 respondents who didn't report an age*

Table S16: Scores split by **education level**

	N	NUM	MAT	CRT	BEL	PRF	REF
No college	771	$-.49_{.04}$	$-.48_{.03}$	$-.38_{.03}$	$-.10_{.03}$	$-.11_{.03}$	$-.24_{.03}$
College	2956	$.05_{.02}$	$.05_{.02}$	$.05_{.02}$	$.01_{.02}$	$.00_{.02}$	$.02_{.02}$
Grad School	678	$.32_{.04}$	$.34_{.04}$	$.23_{.04}$	$.06_{.04}$	$.12_{.04}$	$.19_{.04}$

Note: *This analysis omits 2 respondents who didn't report an education level*

Table S17: Scores split by **income**

	N	NUM	MAT	CRT	BEL	PRF	REF
Under \$30k	1383	$-.19_{.03}$	$-.22_{.03}$	$-.16_{.03}$	$-.02_{.03}$	$-.16_{.03}$	$-.06_{.03}$
\$30k to \$70k	1628	$-.03_{.02}$	$-.05_{.02}$	$-.06_{.02}$	$-.04_{.03}$	$-.03_{.02}$	$-.05_{.02}$
Over \$70k	1377	$.23_{.03}$	$.28_{.03}$	$.22_{.03}$	$.07_{.03}$	$.20_{.03}$	$.12_{.03}$

Note: *This analysis omits 19 respondents who didn't report income.*

Table S18: Scores split by **political affiliation**

	N	NUM	MAT	CRT	BEL	PRF	REF
Democrat	2068	$.01_{.02}$	$.02_{.02}$	$.02_{.02}$	$.12_{.02}$	$-.01_{.02}$	$.04_{.02}$
Republican	1136	$-.12_{.03}$	$-.15_{.03}$	$-.14_{.03}$	$-.33_{.02}$	$-.08_{.03}$	$-.18_{.03}$
Neither	1203	$.10_{.03}$	$.12_{.03}$	$.09_{.03}$	$.10_{.03}$	$.09_{.03}$	$.11_{.03}$

Table S19: Scores split by **survey platform**

	N	NUM	MAT	CRT	BEL	PRF	REF
Prolific	1091	$.37_{.03}$	$.35_{.03}$	$.48_{.03}$	$.50_{.03}$	$.26_{.03}$	$.33_{.03}$
MTurk	1028	$.18_{.03}$	$.03_{.03}$	$.18_{.03}$	$-.16_{.04}$	$-.04_{.03}$	$.19_{.03}$
Qualtrics	1141	$-.25_{.03}$	$-.10_{.03}$	$-.30_{.02}$	$-.15_{.03}$	$-.09_{.03}$	$-.26_{.03}$
Forthright	1147	$-.26_{.03}$	$-.25_{.03}$	$-.31_{.03}$	$-.18_{.03}$	$-.12_{.03}$	$-.23_{.03}$

7. Incremental predictive validity of CRT by demographic group

Tables S20-S26 repeat the main pre-registered test of CRT's incremental predictive validity separately for various demographic subgroups (see specification 2 in Table 3 from the main text). Main scripts in the tables are standardized regression coefficients resulting from regressing latent MAT and observed CRT simultaneously on each of the DVs (the column labels). Subscripts are standard errors. This analysis reveals that the CRT adds predictive validity for all subgroups, but somewhat more for young men.

Table S20: DVs simultaneously regressed on CRT and MAT, segregated by **gender**

		BEL	PRF	REF
Female (*n* = 2234)	Latent MAT	**.20** $_{.04}$	**.12** $_{.03}$	**.19** $_{.04}$
	Observed CRT	**.19** $_{.04}$	**.11** $_{.04}$	**.34** $_{.03}$
Male (*n* = 2138)	Latent MAT	**.21** $_{.04}$	**.08** $_{.04}$	**.15** $_{.04}$
	Observed CRT	**.26** $_{.04}$	**.27** $_{.04}$	**.46** $_{.03}$
Other (*n* = 35)	Latent MAT	-.34 $_{.24}$	**.62** $_{.23}$	.07 $_{.25}$
	Observed CRT	**.60** $_{.21}$	-.36 $_{.24}$	.34 $_{.23}$

Table S21: DVs simultaneously regressed on CRT and MAT, segregated by **age**

		BEL	PRF	REF
Under 35 (*n* = 1454)	Latent MAT	**.15** $_{.05}$	.06 $_{.05}$	.08 $_{.04}$
	Observed CRT	**.27** $_{.04}$	**.25** $_{.05}$	**.49** $_{.04}$
35 to 55 (*n* = 1643)	Latent MAT	**.24** $_{.04}$	**.17** $_{.05}$	**.23** $_{.04}$
	Observed CRT	**.27** $_{.04}$	**.16** $_{.05}$	**.38** $_{.04}$
Over 55 (*n* = 1307)	Latent MAT	**.25** $_{.05}$	.10 $_{.05}$	**.22** $_{.05}$
	Observed CRT	**.17** $_{.05}$	**.19** $_{.05}$	**.33** $_{.04}$

Note: *This analysis omits 3 respondents who didn't report an age*

Table S22: DVs simultaneously regressed on CRT and MAT, segregated by **race / ethnicity**

		BEL	PRF	REF
White (*n* = 3336)	Latent MAT	**.22** $_{.03}$	**.09** $_{.03}$	**.17** $_{.03}$
	Observed CRT	**.24** $_{.03}$	**.23** $_{.03}$	**.41** $_{.03}$
Black (*n* = 365)	Latent MAT	.09 $_{.10}$	.06 $_{.11}$	**.36** $_{.10}$
	Observed CRT	**.20** $_{.10}$	.06 $_{.10}$	.12 $_{.09}$
Hispanic (*n* = 204)	Latent MAT	.21 $_{.12}$	.14 $_{.13}$	.10 $_{.11}$
	Observed CRT	**.25** $_{.11}$	.09 $_{.12}$	**.52** $_{.10}$
Asian (*n* = 193)	Latent MAT	.08 $_{.14}$	.26 $_{.14}$	-.14 $_{.12}$
	Observed CRT	**.34** $_{.13}$	.17 $_{.13}$	**.71** $_{.11}$
Other / multiracial (*n* = 309)	Latent MAT	**.22** $_{.09}$	.16 $_{.10}$	**.20** $_{.09}$
	Observed CRT	**.21** $_{.09}$	.09 $_{.09}$	**.37** $_{.08}$

Note: *The "other" category aggregates those who selected multiple races, those who selected Middle Eastern, Pacific Islander, Other, and those who declined to respond.*

Table S23: DVs simultaneously regressed on CRT and MAT, segregated by **education levels**

		BEL	PRF	REF
No college (*n* = 771)	Latent MAT	**.20** $_{.07}$	**.15** $_{.07}$	**.23** $_{.07}$
	Observed CRT	**.24** $_{.06}$	.08 $_{.07}$	**.31** $_{.06}$
College (*n* = 2956)	Latent MAT	**.19** $_{.03}$	**.10** $_{.03}$	**.16** $_{.03}$
	Observed CRT	**.25** $_{.03}$	**.23** $_{.03}$	**.42** $_{.03}$
Grad. School (*n* = 678)	Latent MAT	**.38** $_{.07}$	.08 $_{.07}$	.10 $_{.06}$
	Observed CRT	**.14** $_{.06}$	**.25** $_{.07}$	**.48** $_{.06}$

Note: *This analysis omits 2 respondents who didn't report an education level*

Table S24: DVs simultaneously regressed on CRT and MAT, segregated by **income**

		BEL	PRF	REF
Under $30k (*n* = 1383)	Latent MAT	**.24** $_{.05}$	.10 $_{.05}$	**.17** $_{.05}$
	Observed CRT	**.28** $_{.05}$	**.18** $_{.05}$	**.45** $_{.04}$
$30k to $70k (*n* = 1628)	Latent MAT	**.19** $_{.05}$	**.09** $_{.05}$	**.19** $_{.04}$
	Observed CRT	**.22** $_{.04}$	**.23** $_{.04}$	**.35** $_{.04}$
Over $70k (*n* = 1377)	Latent MAT	**.23** $_{.05}$	.06 $_{.05}$	**.15** $_{.04}$
	Observed CRT	**.22** $_{.04}$	**.23** $_{.05}$	**.43** $_{.04}$

Note: *This analysis omits 19 respondents who didn't report an income level.*

Table S25: DVs simultaneously regressed on CRT and MAT, segregated by **political affiliation**

		BEL	PRF	REF
Democrat	Latent MAT	**.22 $_{.04}$**	**.11 $_{.04}$**	**.14 $_{.04}$**
(*n* = 2068)	Observed CRT	**.27 $_{.04}$**	**.19 $_{.04}$**	**.44 $_{.03}$**
Republican	Latent MAT	**.20 $_{.05}$**	**.13 $_{.05}$**	**.17 $_{.05}$**
(*n* = 1136)	Observed CRT	**.12 $_{.05}$**	**.17 $_{.05}$**	**.35 $_{.05}$**
Neither	Latent MAT	**.16 $_{.05}$**	.06 $_{.06}$	**.18 $_{.05}$**
(*n* = 1203)	Observed CRT	**.29 $_{.05}$**	**.28 $_{.05}$**	**.43 $_{.04}$**

Table S26: DVs simultaneously regressed on CRT and MAT, segregated by survey **platform**

		BEL	PRF	REF
Prolific	Latent MAT	**.13 $_{.05}$**	**.20 $_{.05}$**	**.20 $_{.05}$**
(*n* = 1091)	Observed CRT	**.24 $_{.05}$**	**.15 $_{.05}$**	**.44 $_{.04}$**
MTurk	Latent MAT	**.24 $_{.06}$**	**.16 $_{.06}$**	**.22 $_{.06}$**
(*n* = 1028)	Observed CRT	**.28 $_{.05}$**	**.25 $_{.06}$**	**.32 $_{.05}$**
Qualtrics	Latent MAT	**.26 $_{.05}$**	-.06 $_{.06}$	**.23 $_{.05}$**
(*n* = 1141)	Observed CRT	.09 $_{.05}$	**.19 $_{.05}$**	**.25 $_{.05}$**
Forthright	Latent MAT	**.24 $_{.05}$**	**.17 $_{.06}$**	**.14 $_{.05}$**
(*n* = 1147)	Observed CRT	**.13 $_{.05}$**	.09 $_{.05}$	**.39 $_{.06}$**

8. Effect of 2nd chance on predictive validity for individual items

Table 4 of the main text examined how giving respondents an opportunity to correct their initial errors would affects predictive validity of the CRT. There, we regressed the scale level scores for each of our DVs simultaneously on both the CRT scores observed initially and those observed after respondents had a 2nd chance to correct initial errors (see, also [1]). Tables S28 & S29 conducts the same analysis for each *item*. Naturally, this more granular analysis largely recapitulates what was shown in Table 4, though it also reveals substantial differences among items. Table S27 shows that the 2nd chance made CRT a better predictor for *all* of the NUM items and half of the MAT items (though the positive effects were all much stronger than the negative effects.)

Table S27: Regression of individual NUM and MAT items on CRT and 2nd chance CRT

		**CRT**	**2nd chance CRT**	**Effect of 2nd chance**
**NUM**	1. 1/20 as decimal	**0.06**	**0.41**	**.35**
	2. 3/5 as decimal	0.01	**0.47**	**.46**
	3. 11/4 as decimal	0.02	**0.43**	**.41**
	4. 0.875 is __ / 8	0.03	**0.46**	**.43**
	5. 1.25 is __ / 4	0.02	**0.49**	**.47**
	6. 0.0375 is 3 / __	**0.15**	**0.25**	.09
	7. 10/3 ÷ 5/9	**0.09**	**0.28**	**.19**
	8. 2 is __% of 5	**0.22**	**0.33**	**.11**
**MAT**	1. Area of grey region	**0.31**	**0.31**	-.00
	2. Mean of 6, 8, & x = 6	**0.20**	**0.44**	**.24**
	3. Angle of y	**0.32**	**0.26**	-.05
	4. Triangle perimeter	**0.29**	**0.24**	-.04
	5. 5/6 of n	**0.17**	**0.42**	**.25**
	6. Side of square	**0.28**	**0.27**	-.01
	7. Diameter of wire	**0.24**	**0.35**	**.11**
	8. Painted wall	**0.14**	**0.42**	**.27**

Note: *The numbers in the first two columns are standardized regression coefficients (all standard errors are ~ 0.03). The third column is simply the differences between those two coefficients (all standard errors are ~ 0.06) Bold numbers are statistically significant (p < 0.05).*

Table S28 shows item level results for our non-mathematical scales. *In general*, providing respondents the opportunity to correct their erroneous first answers made the CRT a much worse predictor of reflection in non-mathematical contexts, as well as a poorer predictor of time and risk preferences, profundity judgments, and beliefs about evolution. To our surprise, it had little effect on the CRT's relation with religiosity and rendered it a somewhat better predictor of beliefs in the paranormal.

Table S28: Regression of BEL, PRF, and REF items on CRT and 2nd chance CRT

		CRT	2nd chance CRT	Effect of 2nd chance
**BEL** Inspirational Quotes	1. Endings are beginnings	**0.19**	-0.06	**-.25**
	2. Create your future	**0.16**	**-0.02**	**-.17**
	3. Shots untaken	**0.20**	**-0.10**	**-.30**
	4. Fear the rain	-0.01	**0.12**	**.13**
**BEL** Bullshit Phrases	5. Flow of actions	**0.16**	**0.17**	.01
	6. Hidden meaning	**0.21**	0.05	**-.16**
	7. Imparts reality	**0.14**	**0.18**	.04
	8. Infinite phenomena	**0.09**	**0.16**	.06
**BEL** Evolution	1. It's just a theory	**0.14**	**0.11**	-.03
	2. Eye is too complex	**0.20**	0.06	**-.13**
	3. Whales are similar to sharks	**0.22**	-0.01	**-.23**
**BEL** Paranormal	1. Spirit leaves body	**0.12**	**0.15**	.02
	2. Loch Ness monster exists	**0.09**	**0.13**	.04
	3. Broken mirrors = bad luck	**0.08**	**0.21**	**.12**
**BEL** Religiosity	1. Believe in god	**0.17**	**0.12**	-.05
	2. How religious	**0.10**	**0.16**	.06
	3. Prayer frequency	**0.15**	**0.13**	-.02
**PRF** Time	1. $3400 vs. $3800 in a month	**0.10**	**0.13**	.03
	2. $100 vs. $140 in a year	**0.26**	**-0.06**	**-.32**
	3. $100 vs. $1100 in ten years	**0.20**	**-0.02**	**-.22**
**PRF** Risk (gains)	4. $60 vs. 1% $5000	**0.04**	**-0.08**	-.12
	5. $100 vs. 3% $7000	**0.10**	**-0.08**	**-.19**
	6. $500 vs. 15% $1,000,000	**0.19**	**0.07**	-.12
	7. $100 vs. 75% $200	0.05	**0.14**	.09
**PRF** Risk (losses)	8. 3% $7000 vs. $100	**0.13**	**-0.12**	**-.25**
	9. 75% $200 vs. $100	**0.08**	**0.07**	-.01
**REF**	1. Complementarity	**0.32**	0.01	**-.31**
	2. Middle of June begins	**0.30**	-0.00	**-.31**
	3. _N_ vs. ING	**0.19**	**0.10**	-.09
	4. Mary's mother	**0.20**	**0.20**	-.00
	5. 9/11 Hi-jackers	**0.14**	0.06	-.08
	6. Minor injuries	**0.21**	-0.02	**-.22**
	7. Linda	**0.32**	**-0.17**	**-.48**
	8. Mugs	**0.13**	0.03	-.10

Note: *For discussion of table entries, see Table S27 note.*

9. Results using a 6-item Reflection Scale

On Prolific (a quarter of our data), a coding error prevented recording of responses to item #6 and item #8 in our REF scale. In the main text, we simply impute responses based on the average solution rate in the other three quarters of the data. Here, we exclude those two items and re-run all analyses from the main text.

The relation between CRT and the Reflection scale is still significantly stronger than the relation between MAT and the Reflection scale. Our main test still finds that observed CRT adds significant incremental validity over and above latent MAT. When we replace observed CRT with latent CRT, latent MAT still becomes a significant suppressor variable. Structural equation modeling still shows that MAT fully explains CRT's relation with Numeracy, while the Reflection Scale still explains CRT's relation with Reflective Beliefs and Reflective Preferences. The relation between CRT and the Reflection scale is still significantly higher than the relation between hinted CRT and the Reflection scale. See Table S29, Figure S11, and Table S30 for details.

Table S29: Simultaneous regression of 6-item REF on CRT and MAT

		REF
**Specification 1:**	Observed MAT	**.13** $_{.02}$
	Observed CRT	**.44** $_{.02}$
**Specification 2:**	Latent MAT	**.17** $_{.03}$
	Observed CRT	**.40** $_{.02}$
**Specification 3:**	Observed MAT	-.05 $_{.03}$
	Latent CRT	**.62** $_{.03}$
**Specification 4:**	Latent MAT	-.07 $_{.04}$
	Latent CRT	**.64** $_{.04}$

Note: *Numbers are standardized regression coefficients. Subscripts are their standard errors. Each specification involves the regression of a 6-item version of REF onto both CRT and MAT simultaneously, using observed scores for REF and either observed or latent scores for the IVs.*

Figure S11: A structural equation model of CRT's relation with our DVs, using a 6-item REF

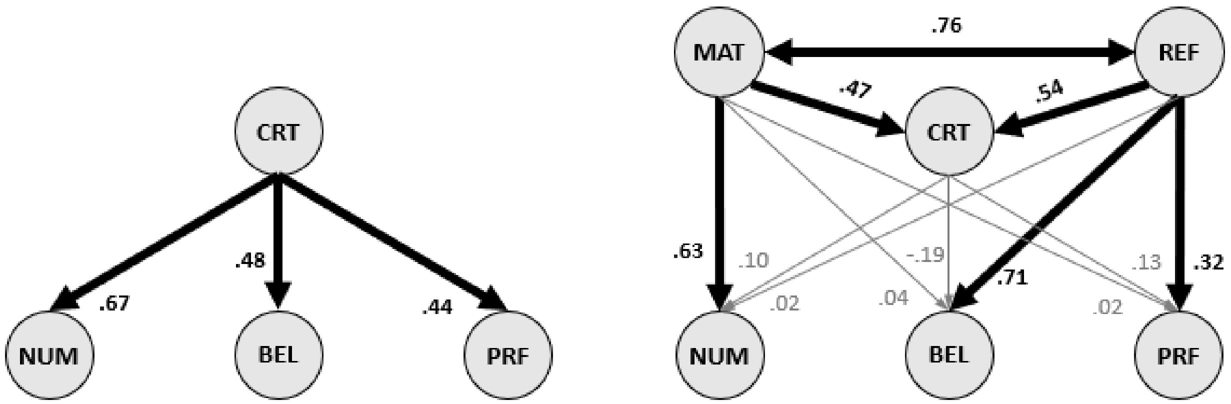

Note: *All variables are latent. Path coefficients are standardized. Bold paths are significant (p < .05).*

Table S30: Simultaneous Regression of REF on CRT and hinted CRT

	REF
CRT	**.47** $_{.03}$
Second chance CRT	**.08** $_{.03}$
Effect of second chance on validity	-.39 $_{.06}$
t-statistic	-7.0

Note: *Numbers are standardized regression coefficients. Subscripts are their standard errors. Presents the regression of a six-item version of REF onto both CRT and Hinted CRT simultaneously, using observed scores for all variables. Hinted CRT score includes items solved before the hints and items solved after the hints.*

10. Results with alternative scoring of the Profundity subscale

As noted in footnote 2, we decided to abide by our pre-registered decision to score our 8-item profundity subscale as the *difference* between profundity ratings of the four "inspirational" (albeit cliché) quotes (items 1 through 4), and the four baroque, non-sensical "bullshit" phrases (items 5 through 8) entailing a prediction that more reflective respondents would have a stronger tendency to penalize word salads for being non-sensical than inspirational quotes for being cliché. And they *do*, but not by much. Higher CRT respondents judged *all* these statements as less profound, which yielded small *differences* between the two *types* of statements. If we had, instead, scored all eight items the same way, scores on our BEL scale would have correlated much more strongly with both CRT scores (0.30 vs. 0.20) and REF scores (0.29 vs. 0.16). Moreover simultaneous regressions of BEL on CRT and MAT would show an increased CRT advantage, while the SEM results would be identical (because they already estimate the reliability maximizing relation between each item and its latent constructs), and invalidating the lures would now slightly decrease CRT's predictive validity. See Table S31 and S32 for details.

Table S31: Simultaneous regression of BEL on CRT and MAT

		BEL
**Specification 1:**	Observed MAT	**.15** $_{.02}$
	Observed CRT	**.30** $_{.02}$
**Specification 2:**	Latent MAT	**.19** $_{.03}$
	Observed CRT	**.26** $_{.03}$
**Specification 3:**	Observed MAT	.02 $_{.03}$
	Latent CRT	**.44** $_{.03}$
**Specification 4:**	Latent MAT	.02 $_{.04}$
	Latent CRT	**.44** $_{.04}$

Note: *Numbers are standardized regression coefficients. Subscripts are their standard errors. Each specification involves the regression of BEL with homogenous profundity scoring onto both CRT and MAT simultaneously, using observed scores for BEL and either observed or latent scores for the IVs.*

Table S32: Simultaneous Regression of BEL on CRT and hinted CRT

	REF
CRT	**.25** $_{.03}$
Second chance CRT	**.18** $_{.03}$
Effect of second chance on validity	-.07 $_{.06}$
t-statistic	-1.2

Note: *Numbers are standardized regression coefficients. Subscripts are their standard errors. Presents the regression BEL with homogenous profundity scoring onto both CRT and Hinted CRT simultaneously, using observed scores for all variables. Hinted CRT score includes items solved before the hints and items solved after the hints.*

Supplementary references

1. Meyer, A. & Frederick, S. The formation and revision of intuitions, *Cognition*, *240*, 105380 (2023).
